# Population pharmacokinetic modelling of imatinib in healthy subjects receiving a single dose of 400 mg

**DOI:** 10.1007/s00280-022-04454-y

**Published:** 2022-07-14

**Authors:** Yi-Han Chien, Gudrun Würthwein, Pablo Zubiaur, Bianca Posocco, María Ángeles Pena, Alberto M. Borobia, Sara Gagno, Francisco Abad-Santos, Georg Hempel

**Affiliations:** 1 Westfälische Wilhelms-Universität Münster, Institut für Pharmazeutische und Medizinische Chemie-Klinische Pharmazie, Corrensstraße 48, 48149 Münster, Germany; 2 Clinical Pharmacology Department, Hospital Universitario de la Princesa, Pharmacology Department, Faculty of Medicine, Universidad Autonoma de Madrid, Instituto de Investigación Sanitaria la Princesa (IIS-Princesa), Madrid, Spain; 3 Centro de Investigación Biomédica en Red de Enfermedades Hepáticas y Digestivas (CIBEREHD), Diego de León 62, 28006 Madrid, Spain; 4 Experimental and Clinical Pharmacology Unit, Centro di Riferimento Oncologico di Aviano (CRO), IRCCS, Via Franco Gallini, 2, 33081 Aviano, PN Italy; 5 Farmacología Clínica, Hospital Universitario Marqués de Valdecilla-Pabellón, 15-planta 2 Avda. Valdecilla s/n., 39008 Santander, Cantabria Spain; 6 Clinical Pharmacology Department, Hospital Universitario La Paz, Pharmacology Department, Faculty of Medicine, Universidad Autonoma de Madrid, IdiPAZ, Madrid, Spain

**Keywords:** Imatinib, Population pharmacokinetics, NONMEM, Ph + leukemia, Empirical Bayesian estimation, MAP Bayesian estimation

## Abstract

**Purpose:**

Imatinib is indicated for treatment of CML, GIST, etc. The population pharmacokinetics (popPK) of imatinib in patients under long-term treatment are reported in literature. Data obtained from bioequivalence trials for healthy subjects were used to evaluate the influence of demographic and pharmacogenetic factors on imatinib pharmacokinetics (PK) in a collective without concurrent drugs, organ dysfunction, inflammation etc. In addition, the differences in PK between the healthy subjects and a patient cohort was examined to identify possible disease effects.

**Methods:**

26 volunteers were administered orally with single dose of 400 mg imatinib. 16–19 plasma samples per volunteer were collected from 0.5 up to 72 h post-dose. The popPK was built and post hoc estimates were compared with previously published PK parameters evaluated by non-compartmental analysis in the same cohort. The predictivity of the model for data collected from 40 patients with gastrointestinal stromal tumors at steady state was evaluated.

**Results:**

The popPK was best described by a two-compartment transit model with first-order elimination. No significant covariates were identified, probably due to the small cohort and the narrow range of demographic covariates; CYP3A5 phenotypes appeared to have some influence on the clearance of imatinib. Good agreement between non-compartment and popPK analyses was observed with the differences of the geometric means/ median of PK estimates below 10%. The model indicated lower clearance for patients compared to healthy volunteers (*p* value < 0.01).

**Conclusion:**

The two-compartment transit model adequately describes the absorption and distribution of imatinib in healthy volunteers. For patients, a lower clearance of imatinib compared to healthy volunteer was estimated by the model. The model can be applied for dose individualization based on trough concentrations assuming no significant differences in absorption between patients and healthy volunteers.

**Supplementary Information:**

The online version contains supplementary material available at 10.1007/s00280-022-04454-y.

## Introduction

Imatinib, marked in its mesylate salt under the name of Gleevec (USA) or Glivec (Europe/Australia) among others, is widely used as a chemotherapeutic agent to treat different forms of cancer, the most prominent being chronic myelogenous leukemia (CML), Philadelphia chromosome-positive acute lymphoblastic leukemia (Ph + ALL) and gastrointestinal stromal tumor (GIST). Being the first tyrosine kinase inhibitor approved on the market, imatinib was introduced into clinical treatment about 20 years ago and is used until now. It is reported to have increased the 8-year survival rate of CML patients from a historical rate of less than 20–87%, [1], and 10-year survival rate to 83.3% [2]. However, the side effect of imatinib, as well as its occasionally low efficiency has often resulted in non-compliance or even interruption of the treatment [3].

Up to date, several pharmacokinetic models of imatinib are reported for patients mainly with CML or GIST [4–15]. Among them, body weight [6, 7, 10, 11, 13] and gender [13] are reported as covariates for both clearance (CL) and volume of distribution (Vd). Age is reported as a covariate for CL [13]. The *ABCB1* C3435T (rs1045642) polymorphism is reported to influence the CL of imatinib [9, 15] and so are the *CYP3A5* A6986G polymorphism [9]. The influence of CYP3A5*3 on CL has been questioned by the study of Yamakawa et al. [15]. The activity of CYP3A4, the enzyme mainly responsible for the metabolism of imatinib, appear to have limited influence on CL, because the drug is only partly metabolized and higher amounts are excreted unchanged through the bile [13, 14].

For healthy individuals, the population pharmacokinetics of imatinib has been studied among Korean volunteers by NONMEM, with age being verified as a covariate for Vd [16]. In the current study, a popPK model for single-dose administration of 400 mg imatinib is evaluated among healthy Caucasian volunteers. Since the data were collected from the two randomized and crossover bioequivalence studies, with the eligibility criteria being set as those established as standard of bioequivalence clinical trials, the impact of demographic as well as genetic factors on the PK of imatinib are analysed with a cleaner patient related background (no concurrent drugs, no organ dysfunction, no inflammation, etc.), so that also smaller effects of genetic polymorphisms on the pharmacokinetics can be identified. With a sampling period of 72 h and frequent sampling around predicted *t*_max_ (Fig. 2), we are also able to have a clearer look into imatinib PK. The PK estimates are compared with previously published non-compartmental analysis (NCA) of the same data [17] and the PK between healthy volunteers and patients is compared by testing the predictivity of the model to patients at steady state.

## Materials and methods

### Study population for popPK model

Pharmacokinetic data were obtained from healthy volunteers (8 females and 18 males), involving 472 observations, who have participated in the two randomized crossover bioequivalence studies. Eligibility criteria were those established as standard in bioequivalence clinical trials: healthy subjects, as determined by medical history and physical examination and subjects have accepted a subsequent pharmacogenetic test [17]. Imatinib was administered orally with one single dose of 400 mg. The subjects with a BMI within 18–30 kg/m^2^, and all the clinical laboratory values within normal reference ranges, or judged as within acceptable deviations by investigators, were included. The demographic characteristics of study population are summarized in Supplement Table S1.

Clinical trials were performed in Hospital Universitario de La Paz, Madrid and Hospital General de Alicante, Spain. All of them were approved by the respective Research Ethics Committee of each hospital and were duly authorized by the Spanish Medicines Agency (AEMPS); the guidelines on Good Clinical Practices were fully applied, complying with current Spanish legislation on clinical research in humans and with the Declaration of Helsinki. All healthy volunteers provided informed consent for the clinical trial and for research involving pharmacogenetics.

### Sampling and analysis

16 to 19 blood samples were collected between 0.5 and 72 h post-dose from each healthy volunteer (12–16 samples within 0.5–12 h post-dose and 2–3 samples 24, 48, 72 h post-dose, see also Fig. 2).

The HPLC–MS/MS method used to quantify imatinib in healthy subjects’ samples was fully validated and complied with the European Medicines Agency (EMA)’s requirements [18], meeting the regulatory standards for its use in a bioequivalence trial. The validation included a comprehensive check of the accuracy, precision, repeatability, specificity, limit of quantification (10 µg/L), limit of detection, and linearity range of the method [17].

As for the pharmacogenetic analysis, blood samples were collected for DNA extraction and a total of 18 alleles was genotyped: *CYP3A4* (*20, *22), *CYP3A5* (*3), *CYP2C9* (*2, *3), *CYP2C19* (*2, *3, *17), *CYP2C8* (*2, *3, *4), *CYP2B6* G516T (rs3745274), *CYP2D6* (*3, *4, *5, *6, *9), and *ABCB1* C3435T (rs1045642) (for details see [17] and the supplement). All genotype frequencies comply with the Hardy Weinberg equilibrium rule and no differences were observed in allele frequencies compared to the expected [17]. The genotypes of the study group are presented in Supplement Fig. S1.

### Software

NONMEM^®^ (version 7.4.3), Pirana (version 2.9.9), PsN (version 5.0.0), Xpose (version 4.7.1), and R (version 4.0.4) were used for popPK analysis and model diagnostics. Data preparation and statistical analysis were performed with R.

### Modelling statistics

First-order conditional estimation (FOCE) with interaction was used for popPK modelling. Inclusion of inter-individual variability (IIV) was tested by exponential models and described by coefficient of variation (CV%) [19]. Additive, proportional and combined error models were tested for estimating the residual variance (equations see VAR.3 in [20]).

### Structure model development

The workflow published by Byon et al. [21] was followed during model development. To describe the distribution of imatinib, one-, two- and three-compartment distribution systems were tested aligning with first-order elimination and different absorption hypotheses. As absorption models, (i) first-order absorption, (ii) zero-order absorption—all models with or without lag time, (iii) double peak models with two independent first-order absorption processes, as well as (iv) transit models were tested, respectively. The transit models were constructed according to Savic et al. [22]: multiple transit compartments are to be passed by the substance after administration until it reaches the depot compartment for the further absorption process through first-order kinetics into the blood circulation.

Model selection was based on the likelihood ratio test (*p* value = 0.01) for nested models, the Akaike information criterion (AIC) for non-nested models and goodness-of-fit (GOF) plots. Only popPK models with successful completion of the covariance step were considered [21].

### Covariates analysis

Scientific merits of each potential covariate were assessed based on mechanistic plausibility and prior scientific knowledge before covariate analysis. The impact of demographic and genetic covariates on the PK of imatinib were tested by forward selection (*p* value = 0.05) followed by backward elimination (*p* value = 0.01) [23]. Body weight, BMI, and BSA were tested as continuous covariates, while gender as categorical covariate for both CL and V1. The genetic factors were tested as categorical covariates for CL and *ABCB1* C3435T genotype additionally for Ka. Categorical covariates were tested using a proportional shift model [24]. When estimating the impact from CYP3A4, CYP2C8, CYP2C9, CYP2C19, and CYP2D6 on CL, samples were pooled together according to their reported phenotypes to increase the power of the analysis: *CYP3A4* (NMs (*n* = 22): *1/*1 vs. IMs (*n* = 4): *1/*20, *1/*22, *22/*22) [25], *CYP2C8* (*1/*1 (*n* = 18) vs. *1/*3 (*n* = 3) vs. *1/*4, *4/*4 (*n* = 5)) [26], *CYP2C9* (NMs (*n* = 16): *1/*1 vs. IMs (*n* = 10): *1/*2, *1/*3, *3/*3) [27], *CYP2C19* (IMs (*n* = 9): *1/*1 vs. UMs (*n* = 10): *1/*17 vs. IMs (*n* = 6)/ PMs (*n* = 1): *1/*2, *2/*2) [28] and *CYP2D6* (NMs (*n* = 14): *1/*1 vs. IMs (*n* = 11)/ PMs (*n* = 1): *1/*3, *1/*4, *1/*5, *4/*5) [29]. Pharmacogenetic phenotype was inferred according to Clinical Pharmacogenetics Implementation Consortium (CPIC) guidelines when available. The impact of continuous covariates was assessed by linear, hockey-stick (using median as break point), exponential and power functions [24]. Body weight was additionally tested by allometric function [30].

### Model evaluation

Visual predictive check (VPC, *n* = 1000 simulations) and bootstrap analysis (*n* = 1000 replications) were used for internal model evaluation.

### Empirical Bayesian estimates from popPK vs. non-compartment analysis

Empirical Bayesian estimates (EBE) of the final popPK model were compared with estimates of NCA [17] derived in the same dataset using paired *t* test.

### Application of the final popPK model to patient data

The predictivity of the final popPK model derived from healthy volunteers for patients under treatment with imatinib at steady state was tested: data from 41 patients (187 plasma samples) treated for GIST were provided by Experimental and Clinical Pharmacology Unit, Centro di Riferimento Oncologico di Aviano (CRO) IRCCS, Aviano, Italy. The LC–MS/MS method used to quantify patients’ plasma samples was validated according to “EMA guideline on bioanalytical method validation” [18] and “FDA Bioanalytical Method Validation – Guidance for Industry” [31] with an intra- and inter-day precision of ≤ 4.7% and ≤ 7.1%, respectively, and the intra- and inter-day accuracy being between 92.7–104.7% and 99.5–103.8%, respectively.

The patients were prescribed with imatinib at different doses (200, 300, 400, 600 mg q.d., and 400 mg b.i.d.). All the patients were recorded being at steady state, and most of the plasma samples were collected at trough levels.

Based on the final popPK model, the maximum a posteriori (MAP Bayesian) estimation was used (MAXEVAL = 0 in $ESTIMATION) to calculate the individual predictions (IPRED) and CL for the patients. The prediction error (PE) of each plasma observation, as well as individual PK estimates of CL were calculated according to the following equations, respectively [32, 33]:

1 $${PE}_{DV}=\frac{(IPRED-DV)}{DV}\times 100\%,$$

2 $${PE}_{CL}=\frac{({CL}_{pop}-{CL}_{bayesian})}{{CL}_{bayesian}}\times 100\%,$$

with DV observed plasma concentration, CL_pop_ the a priori predicted and CL_bayesian_ Bayesian maximum a posteriori estimated individual CL. The median and the median absolute value are derived as bias and precision, respectively, to evaluate the predictions of plasma concentration and CL [32].

## Results

### PopPK model for healthy volunteers

A total of 472 plasma observations from 26 healthy volunteers was included for model construction, with no plasma level being below the LOQ of the analysis method. The median age, body weight, BMI and body surface area (BSA) [34] were 23.0 years (19.7–31.0), 69.5 kg (52.0–96.0), 22.5 kg/m^2^ (20.0–30.0), and 1.86 m^2^ (1.52–2.22), respectively ([17] and Supplement Table S1). The detailed information about the model development process is listed in Table 1. Aligning with first-order absorption, a two-compartment distribution system showed notable improvement in both GOF plots and OFV when comparing to a one-compartment model (Table 1, model A2 vs. A1, dOFV = − 88.55). A three-compartment model showed no further advancement. Similar results regarding the number of distribution compartments were observed when testing other absorption models. For imatinib, a delayed absorption process was observed. Models of different absorption hypotheses were improved by an additional parameter describing the lag time (Table 1, models B1–B4). Further improvement was then obtained by a fourth IIV-parameter for this lag process (Table 1, models C1–C5). Based on that construction (two-compartment model with four IIV-parameters), different absorption models were tested. Compared with the first-order absorption model, the zero-order absorption model (**C2**, dAIC = − 92.88) and the double peak model with a second fixed absorption process (**C3**, dAIC = − 34.42) were superior. Because of the small difference between the two absorption rates (Ka1 = 1.03 h − 1, Ka2 = 0.996 h − 1), the double peak model was further simplified by defining Ka1 = Ka2 for the comparison (**C4**, dAIC = − 45.48). The transit absorption model modelling the delayed absorption with a mean transit time (MTT) rather than a lag time best described the dataset (**C5**, dAIC = − 111.55).

**Table 1:** The process of structure model development

No. Model	IIV on	OFV	AIC
A: Base-compartment model			
A1: One-compartment model with first-order absorption	CL V1 Ka	5833.20	5849.20
A2: Two-compartment model with first-order absorption	CL V1 Ka	5744.65	5764.65
A3: Three-compartment model with first-order absorption	CL V1 Ka	5744.52	5766.52
B: Introduction of lag time, different absorption models			
B1: Two-compartment model with first-order absorption (with lag time)	CL V1 Ka	5598.49	5618.49
B2: Two-compartment model with zero-order absorption (with lag time) (1)	CL V1 D	5551.73	5571.73
B3: Two-compartment double peak model (two different Ka & lag times) (2)	CL V1 Ka1	5406.26	5582.43
B4: Two-compartment transit model	CL V1 Ka	5570.73	5592.73
C: Introduction of additional IIV			
C1: Two-compartment model with first-order absorption (with lag time)	CL V1 Ka ALAG	5458.12	5480.12
C2: Two-compartment model with zero-order absorption (with lag time) (1)	CL V1 D1 ALAG	5365.24	5387.24
C3: Two-compartment double peak model (two different Ka & lag times)	CL V1 Ka1 ALAG1	5417.70	5445.70
C4: Two-compartment double peak model (one ka & two different lag times)	CL V1 Ka ALAG1	5408.64	5434.64
C5: Two-compartment transit model	CL V1 Ka MTT	5344.57	5368.57
D: Introduction of OMEGA BLOCK			
D1: Two-compartment transit model with OMEGA BLOCK between IIV on CL and Ka	CL V1 Ka MTT	5300.04	5326.04

Models listed above were constructed with proportional error model, besides A1, which fit better with a combined error model. (1) Unstable model: PK estimates were influenced by initial values; (2) Unrealistic PK estimates

*AIC* Akaike information criterion; *ALAG* absorption lag time; *CL* clearance; *D* absorption duration for central compartment; *IIV* inter-individual variability; *Ka* absorption rate constant; *MTT* mean transit time; *OFV* objective function value; *V1* central volume of distribution

As a result, a two-compartment transit model with first-order elimination was set as the structure model. A proportional error model was selected, since it stabilized the two-compartment models from being dependent on the initial values. IIV was estimated for CL, central volume of distribution (V1), Ka and MTT, respectively. Due to the strong correlation between the IIVs of CL and V1 (*r* > 0.9), an OMEGA BLOCK was added to these two PK parameters at the end of the model development process (**D1**). The shrinkages of the evaluated inter-individual random effects are all well below 20%.

Based on the developed structural model, the covariate analysis was conducted. The relationship between the genotypes and the corresponding inter-individual deviations from the population mean (ETA) of the PK parameters is shown in Supplement Fig. S1. None of the covariates improved the PK model during forward inclusion, besides *CYP3A5* (*1/*3 (IMs) vs. *3/*3 (PMs), which was included as a covariate on CL (dOFV: − 3.95, *p* value: 0.047). A first hint for a lower CL for individuals carrying *1/*3 (IMs) compared to the genotype of *3/*3 (PMs) was shown by PK estimates. However, with only 4 subjects compared with the remaining 22, the power of this test is too small to be valid. A further covariate analysis of *CYP3A5* genotypes is thus considered as meaningless and was not conducted.

**Fig. 1:**
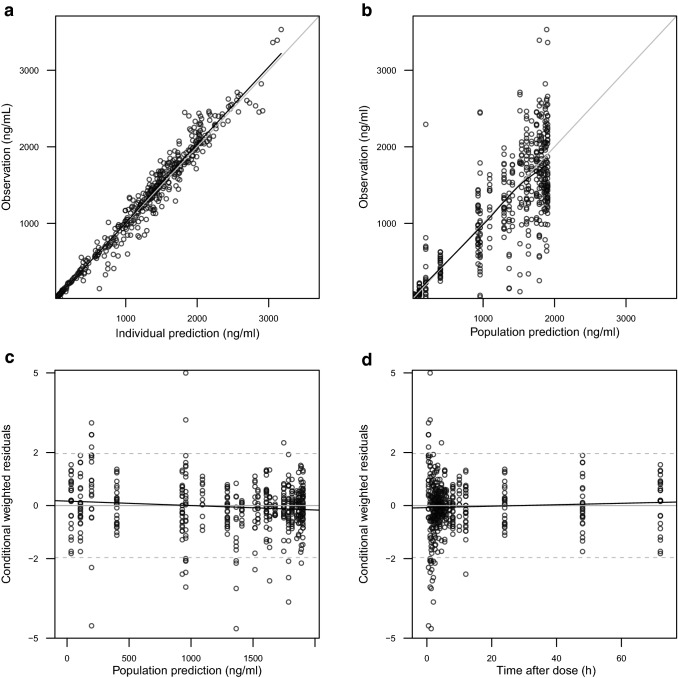
GOF plots of the final popPK model of imatinib. Upper panel, observed imatinib plasma concentrations versus individual predictions (**a**) and population predictions (**b**); lower panel, conditional weighted residuals versus population predictions (**c**) and time after dose (**d**)

As a result, the final popPK model is constructed without any covariate. According to the GOF plots (Fig. 1), no systematic bias or error could be identified. The PK estimates of the model, as well as the corresponding relative standard errors (RSEs) are summarized in Table 2.

**Table 2:** Final estimates of population PK parameters

Parameter	Description	Estimate	%RSE	1000 Bootstrap replicates (93.6% successful) Median (95% CI)
CL/F (L/h)	Apparent oral clearance	13.2	5.0	13.1 (11.9–14.5)
Q/F (L/h)	Intercompartmental clearance	3.75	20.5	3.81 (2.78–5.62)
V1/F (L)	Apparent volume of central compartment	172	4.7	170 (154–189)
V2/F (L)	Apparent volume of peripheral compartment	43.6	10.4	44.0 (37.4–54.1)
Ka (h^−1^)	Absorption rate constant	1.22	18.2	1.20 (0.829–1.84)
MTT (h)	Mean transit time (of the absorption transit compartments)	0.537	14.9	0.530 (0.376–0.735)
N	Number of transit compartments	3.62	12.1	3.60 (2.51–18.3)
IIV_CL_ (%)	Interindividual variability of CL	24.8	12.8	24.2 (18.3–30.3)
IIV_V1_ (%)	Interindividual variability of V1	27.7	11.4	27.3 (19.9–33.7)
IIV_Ka_ (%)	Interindividual variability of Ka	88.3	15.7	85.1 (52.6–128.6)
IIV_MTT_ (%)	Interindividual variability of MTT	80.5	17.3	77.4 (40.2–122.2)
Prop error (%)	Proportional error	13.6	10.0	13.2 (10.6–15.8)

*RSE* relative standard error

### Internal evaluation

**Fig. 2:**
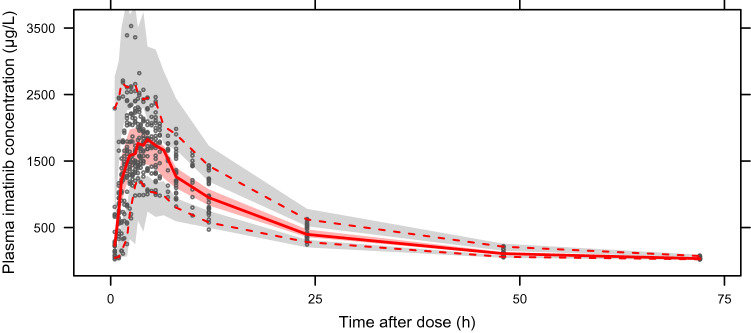
VPC plot of the final popPK model of imatinib. Visual predictive check (1000 simulations) of the final popPK model for a single oral administration of 400 mg imatinib. The black circles represent the observed imatinib plasma concentrations. The median (solid red line), 5th percentile (lower dashed red line) and 95th percentile (upper dashed red line) of the observations per bin are presented. The 95% confidence intervals of the simulations are presented for median (red area), 5th percentile (lower gray area), and 95th percentile (upper gray area) on the plot, respectively

As shown in Table 2, all estimates of our popPK model are within the 95% CIs of the medians of the bootstrap analysis. The VPC plot (Fig. 2) indicates good simulation properties of the model. The median, 5th and 95th percentiles of the plasma observations are all within the corresponding simulated 95% CI ranges. No evidence of model misspecification is indicated.

### Empirical Bayesian estimates from popPK vs. non-compartment analysis

Based on the final popPK model, individual estimates were derived and compared with results of NCA analysis of the same dataset. In Table 3, the geometric means of log-normal distributed parameters, including AUC_0-72_, *C*_max_, *V*_*d*_ (central plus peripheral Vd for the popPK model) and CL, as well as the median of *t*_max_ from both approaches are listed. Apart from *t*_max_ and AUC_0-72_, all the other PK estimates from the two approaches are significantly different (paired *t* test, *p* value < 0.05), although the differences of their geometric means/ median of PK estimates are below 10%. Geometric mean values of *C*_max_ and *V*_*d*_ are slightly higher by NCA, whereas individual CL is slightly lower estimated by NCA (Table 3).

**Table 3:** Comparison of PK estimates: NCA vs. popPK model

		NCA (Pena et al. [17])		Present popPK model	*p* value (paired *t* test)
x̅_G_ (RSD_G_)	AUC_0-72_		31.2 (29.0%)	30.2 (24.5%)	0.116^(1)^
	C_max_		1.96 (27.8%)	1.84 (26.6%)	< 0.05^(1)^
	Vd		236 (23.9%)	216 (21.9%)	< 0.01^(1)^
	CL		12.5 (29.3%)	13.2 (25.0%)	< 0.01^(1)^
x̃(IQR)	t_max_		3.04 (2.54–4.38)	2.97 (2.50–4.13)	0.881

x̅_G_: geometric mean; x̃: median; RSD_G_: geometric relative standard deviation; IQR: interquartile range; *AUC*_*0-72*_ area under the plasma concentration versus time curve from time zero to 72 h post-dose; *Cmax* maximum observed plasma concentration; *NCA* non-compartmental analysis; *t*_*max*_ time to maximum observed plasma concentration (1) comparison of log-transformed data

### Application of the final popPK model to patient data

In the external dataset from patients treated with imatinib, one of the 41 patients was excluded due to reported non-compliance (seven samples). Furthermore, observations below the LOQ were excluded (two samples). The demographic characteristics of the patient cohort are summarized in Supplement Table S1. Based on the remaining 178 imatinib plasma observations, the predicted plasma levels as well as individual CL values of the patient cohort were estimated by the final popPK model of the healthy volunteers. The MAP Bayesian estimation was used when calculating the individual PK parameter for patients. As the popPK model does not include any covariate for CL, the value for CL_pop_ is equal to 13.2 L/h when calculating PE_CL_. Considering the difficulty in following the treatment schedule of an outpatient treatment, the observations that were recorded exceeding their intended, prescribed dosage intervals (24 h for q.d. and 12 h for b.i.d.) were excluded to build a subset of data as the second analysis group. Thus we accounted for possible non-adherence and the uncertainty of the exact dosing time points of patients before their next observation visits. For all data as well as the data within one dosing-interval, bias and precision were calculated (Table 4).

**Table 4:** Application of the final popPK model from healthy volunteers to patient data

	All data		Data within one dosing-interval^a^	
	PE_DV_ (*n* = 178)	PE_CL_ (*n* = 40)	PE_DV_ (*n* = 85)	PE_CL_ (*n* = 35)
Bias (%)	4.6	33.7	− 1.9	27.0
Precision (%)	17.6	36.6	15.2	30.0

Bias: median prediction errors; Precision: median absolute prediction errors. PE_DV_: prediction error of plasma concentration (Eq. (1)) PE_CL_: prediction error of clearance (Eq. (2))

^a^Subset of data to minimize the uncertainly of time of drug administration in outpatient treatment of the GIST-patients

The popPK model can well predict the plasma concentrations of imatinib included in the clinical dataset, with bias and precision estimated as 4.6% and 17.6%, respectively. These two values further decrease to − 1.9% and 15.2%, respectively, when estimating only the subset of data within one dosing interval. Furthermore, no systematic deviation was observed among different doses (300–600 mg q.d. & 400 mg b.i.d.). The bias (6.0%) and precision (18.5%) of observations with 400 q.d. only (*n* = 157) are very close to the results of all data.

The MAP Bayesian individual CL estimates appears to deviate from the population value of our model, with a high bias (33.7%) and a low precision (36.6%) being calculated. When analysing the subset of data, both parameters remain high (Table 4). This indicates that, comparing with the population value, the CLs of the patient cohort were generally lower estimated by the model according to the observed plasma levels (two-sided Mann–Whitney *U* test: *p* < 0.01). In Supplement Fig. S2, it is obvious that the prediction errors of both testing groups are for plasma levels centred around zero while for CL shifted upwards.

## Discussion

### popPK model for healthy volunteers

In the cohort of healthy volunteers, a transit-compartment model best described the observed delayed absorption pattern of imatinib. In contrast to a one-compartment distribution system used in most of the published PK models to describe the distribution of imatinib, a two-compartment model showed a much better fit to the present dataset. Since this improvement became obvious on GOF plots with the observations that were sampled after 48 h post-dose, an extended sampling time up to 72 h post-dose of the dataset have had surely supported this result.

With V1 and V2 estimated by our model to be 172 (IIV ~ 28%) and 43.6 L, respectively, imatinib appears to have a high tissue penetration in human body. It appears that imatinib can cross physiological membranes due to the lipophilic properties at pH 7.4 [35]. By having the unbound imatinib readily cross the capillary wall into tissues, the bound imatinib in the blood vessels is forced to dissociate from the plasma proteins to maintain the equilibrium environment, which would then further advance the distribution of imatinib [36]. Unfortunately, the possible impact of changed plasma protein levels on the PK of imatinib, in view of its high affinity to plasma proteins [37], cannot be studied in our collective of healthy subjects (plasma proteins in normal ranges). This might lead to some deviations when applying our popPK model to patients, who might have abnormal levels of plasma proteins.

Interestingly, the population CL value of our model (13.2 L/h) is very similar to the value estimated among Korean volunteers (13.6 L/h) [16], indicating a negligible racial difference of imatinib CL.

For Covariate Analysis age was not considered because of the small range: 19.7–31.0. *ABCB1* C3435T genotype was tested on CL and Ka. This was under the consideration that ABCB1 (P-glycoprotein), as an efflux transporter of imatinib, is highly expressed in intestinal epithelium and liver cells, influencing absorption and biliary elimination by pumping out xenobiotics [38]. All other genotypes were tested as potential covariates of CL because of their potential involvement in the metabolism of imatinib [39–41]. None of the potential demographic covariates (body weight, BMI, BSA and gender) showed a statistically significant association with the individual PK parameters. This is probably due to the small range of covariates among the healthy subjects, and has to be taken into consideration when applying our popPK model to a clinical setting. Among genetic factors, only the *CYP3A5* genotypes (*3/*3 and *1/*3) improved the fit of the model slightly (*p* value < 0.05). However, due to the insufficient sample size, this result is only interpreted as a trend of *CYP3A5* being a genetic covariate of CL, which is in accordance with the finding by Adeagbo et al., that CYP3A5*3 have significant influence on the CL of imatinib [9]. The observed trend of influence from *CYP2B6* G516T genotypes on CL and *ABCB1* C3435T genotypes on Ka (Supplement Fig. S1) could not be verified. Again, these results could, however, be due to the insufficient sample size.

Pena et al. [17] found a significant difference in Vd between individuals carrying *CYP3A4* *20, *22 alleles in comparison to *1/*1 carriers, and *CYP2B6* G516T (rs3745274) carriers (IM`s) showed a shorter half-life compared to GG, using NCA analysis of the same dataset. In popPK analyses, the impact of phenotypes is primarily tested on the CL, because CL is primary pharmacokinetic parameter and half-lives are derived parameters. Based on the final estimates of our model, only the relation between Vd and *CYP3A4* genotypes can be verified according to a two-sample *t* test (*p* value < 0.01). Overall, it appears that larger investigations are necessary to quantify the effects of genetic polymorphisms on the pharmacokinetics of imatinib. However, the analysis of Pena et al. [17] and our analysis shows that there are no substantial effects of the genotypes on the pharmacokinetics of imatinib.

Among patients, high inter-individual variation with regard to the absorption process of imatinib was described [4, 6, 8, 11]. It has to be emphasized that this high variability is already present within healthy subject, who were administered only a single dose of imatinib within a clinical bioequivalence trial. High IIVs were estimated by our model on Ka and MTT (IIV_Ka_ ~ 88%, IIV_MTT_ ~ 81%). Individual cases with high (3531 µg/L) and low (1073 µg/L) C_max_ were observed during analysis, with about doubled and half of the population median value, respectively. Considering that a plasma trough concentration above 3180 µg/L is associated with a higher frequency of adverse events [42], this individual variation may cause non-adherence to treatment. The reason behind these variations remains unexplained from our study and needs further investigation. However, this may hint at that the variability of the absorption process of imatinib might have not only associations with laboratory values, which were in a normal range within the cohort of healthy volunteers, but also with pharmacogenetic factors. In this study only *ABCB1* C3435T (CC, CT, TT) were tested as covariates for Ka. Although no significant improvement of the model could be obtained from that, there was still a tendency that the wild-type might result in a lower Ka (Supplement Fig. S1). The high variability observed supports the application of therapeutic drug monitoring for imatinib.

### Empirical Bayesian estimates from popPK vs. non-compartment analysis

NCA relies on individual observed data only and is thus very dependent on the density of the sampling time points. In our case, the frequent sampling during the first five hours with individually 8–12 sampling time points has greatly benefited the estimation of NCA, especially for t_max_ and Cmax.

During nonlinear mixed effects modelling, fixed as well as random effects are estimated based on the dataset for all patients. Thus, slight differences of PK estimates are expected between these two analysis approaches [43, 44].

Besides that, in the present study, a slightly higher estimated Cmax is expected by NCA, when some of the observed Cmax could not be reached by the popPK model. A pharmacokinetic model can ideally represent the “true” plasma concentrations, whereas *C*_max_ and *t*_max_ values from NCA contain measurement and documentation errors.

The estimates of *t*_max_ and AUC_0–72_ from the two analysis approaches are statistically not different from each other (Table 3). On one hand, with similar median values calculated from the two approaches, this confirms the population value of t_max_ for the volunteers being at about 3 h post-dose. On the other hand, the popPK model is thus proved able to describe the PK profiles, especially the absorption process of imatinib accurately and precisely. In the bioequivalence studies, the N-desmethyl metabolite was not analysed. Thus, the PK and the AUC of this pharmacologically active metabolite, which accounts for approximately 10% that of the AUC for parent molecule [39], cannot be estimated.

Differences of the geometric means for the other PK parameters are below 10% and do not appear to be clinically relevant. One can expect that the deviations between the popPk and the NCA would increase with decreasing number of sampling time points.

### Application of the final popPK model to patient data

To evaluate the adaptability of our popPK model to patients, the model was applied to a clinical dataset of 40 patients with GIST following long-term treatment. With the bias and precision being under 5% and 20% (Table 4), respectively, the popPK model precisely and accurately estimated the plasma concentrations for patients. Meanwhile, different doses of imatinib (200, 300, 400, 600 mg q.d. & 400 mg b.i.d.) also seem to be well adapted by the model, which on the other hand confirms the linear pharmacokinetics of imatinib. Some plasma levels in the patient cohort were abnormally low and were estimated significantly higher by our model (data not shown). The reason behind is unknown, but the possibility of unrecorded non-compliances or vomiting after drug administration are probable explanations.

Considering that most of the plasma observations involved in this analysis were collected shortly before the next administration, the estimation of the absorption and distribution processes related with the individual estimations of Ka, MTT and V1 of our model, could not be supported by a sufficient amount of data. Therefore, the focus was on the estimate of individual CL values. According to the model, it is assumed that the patient cohort suffering from GIST had a lower CL than the healthy cohort.

In the literature, the reported median CL for patients is ranging between 7.29 and 17.2 L/h [4–15] (Supplement Table S2). In the present study, the median CL for the healthy cohort (13.2 L/h) and for the patients with GIST (9.9 L/h) are both within that range.

However, a decrease in CL over time is reported for imatinib in two investigations [7, 10]. According to Judson et al., the CL of imatinib is though decreased at 29 days of the treatment but raises again in the extension phase [7]. Yet since the model describes the absorption process with only fixed effect [7], it is not sure whether the increased CL in the extension phase is biased by raised absorption problems among patients [45].

In our cohort, the healthy volunteers received one imatinib dose only. Thus, we could not address the question of potential CL-changes after long-term treatment in volunteers as reported in patients. We, therefore, cannot clarify whether the lower CL in patient is a result of the long-term treatment or whether there are further differences in the PK of imatinib between patients and healthy volunteers. Possibly, this might be a long-term side effect of imatinib due to its hepatotoxicity [46], but it could also be influenced by the chemotherapy, other co-medications, or disease progression. As described above, differences in plasma protein binding of imatinib between healthy subjects and patients may—in part—explain the observed differences in the two population studies here. Besides that, to verify whether this decreased CL should lead to over-accumulated imatinib in patients, further PK analysis with regard to changes of the other PK parameters, as well as PD analysis should be conducted.

In summary, a transit model to characterize imatinib PK in healthy subject was established with no significant covariate being found. There is, however, a trend that CYP3A5*3 could have an impact on individual CL for imatinib. High IIVs with regard to the absorption process were not only reported in patients [4, 6, 8, 11] but also observed in our cohort of healthy volunteers, who were administered only a single dose of 400 mg imatinib in strictly controlled and observed bioequivalence clinical trials. This may hint at a potential association between absorption rate and demographic or genetic factors. Regarding that, caution should be urged in clinical treatment of imatinib to maintain efficient clinical response and prevent adverse events.

When applying to trough concentrations of patients being under long-term treatment of imatinib, our popPK model shows good predictability, with generally lower CL being estimated compared to the healthy cohort. For the purpose of dose individualisation, our model can be used to calculate the AUC from trough concentrations under the assumption that absorption does not differ significantly between patients and healthy volunteers.

## Supplementary Information

Below is the link to the electronic supplementary material.

Supplementary file1 (DOCX 160 kb)

## Data Availability

The datasets generated during and/or analysed during the current study are available from the corresponding author on reasonable request.

## Code availability

The NONMEM-code of the final popPK model is available from the corresponding author on reasonable request.
