# Supplementary material for: Population pharmacokinetic modelling of imatinib in healthy subjects receiving a single dose of 400 mg
Source: Cancer Chemother Pharmacol. 2022 Jul 14;90(2):125–36. doi: 10.1007/s00280-022-04454-y (PMC9360108; doi:10.1007/s00280-022-04454-y)
Supplement: Supplementary file 1 — Supplementary file1 (DOCX 160 kb) [file 280_2022_4454_MOESM1_ESM.docx]

**Clinical Pharmacology and Therapeutics**

# **Supplementary Information**

**Population pharmacokinetic modelling of imatinib in healthy subjects with single dose of 400 mg**

**Authors:** Yi-Han Chien, Gudrun Würthwein (ORCID: 0000-0002-7617-183X), Pablo Zubiaur, Bianca Posocco, María Ángeles Pena (ORCID: **0000-0003-0066-8943)**, Alberto M. Borobia (ORCID: **0000-0002-8584-3263),** Sara Gagno, Francisco Abad Santos (ORCID: **0000-0002-6519-8885)**, Georg Hempel^*^ (ORCID: **0000-0002-5790-6423)**

*corresponding author

Westfälische Wilhelms-Universität Münster

Institut für Pharmazeutische und Medizinische Chemie

- Klinische Pharmazie -

Corrensstraße 48

48149 Münster, Germany

E-Mail: georg.hempel@uni-muenster.de

**Genotyping:**

As for the pharmacogenetic analysis, blood samples for DNA extraction were obtained in a EDTA K2 tube and stored at 4 °C. A total of 18 alleles was genotyped for the 26 subjects. The detailed information about the test has been published by Pena et al. [1]. The pharmacogenetic test was done by the Pharmacogenetics Unit of Hospital Universitario de La Princesa in Madrid, Spain. The blood samples were shipped there refrigerated and were analysed by quantitative real-time polymerase chain reaction (qPCR). An automatic DNA extractor (MagNa Pure® System, Roche Applied Science, Indianapolis, Indiana, United State) and a spectrophotometer (NanoDrop® ND-1000 Spectrophotometer, Wilmington, Delaware, United State) were used to extract and quantify DNA. A LightCyler® 2.0 instrument (Roche Diagnostics, Mannheim, Germany) was used to genotype CYP2C8 (*2, *3, *4), CYP2C9 (*2, *3), CYP2C19 (*2, *3, *17), and CYP2D6 (*3, *4, *5) with the probes designed by TIB MOLBIOL (GmbH, Berlin, Germany). Meanwhile, a StepOne^TM^ instrument (Applied BosystemsStep One^TM^ Real-Time PCR System, Foster City, California, US) was used with TaqMan® allele discrimination probes (Applied Biosystems, Foster City, California, US) to genotype CYP2B6 G516T (rs3745274), CYP2D6 (*6, *9), CYP3A4 (*22), CYP3A5 (*3) and ABCB1 C3435T (rs1045642). CYP3A4 (*20) was genotyped using the SNP KASPar genotyping system (LGC Genomics. Herts, UK), with the ABI PRISM 7900HT sequence detection system (Applied Biosystems, Darmstadt, Germany) being used for fluorescence detection and allele assignment. All carriers of CYP3A4 (*20) were confirmed by Sanger sequencing in an ABI PRISM 3700 DNA Analyzer capillary sequencer (Applied Biosystems, Foster City, California, US).


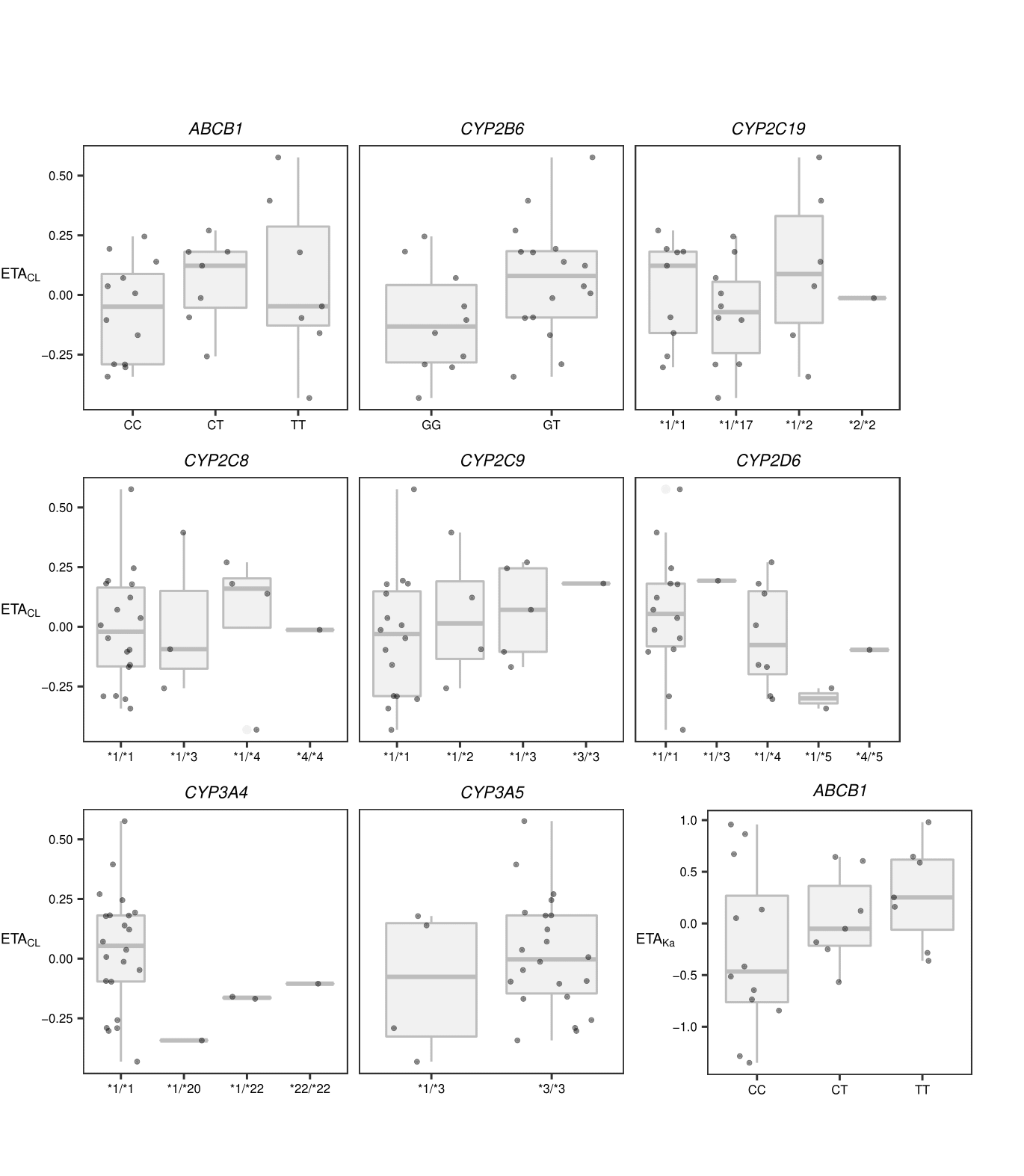


**Supplement Fig. S1** Individual variations (ETA) vs. Genotypes


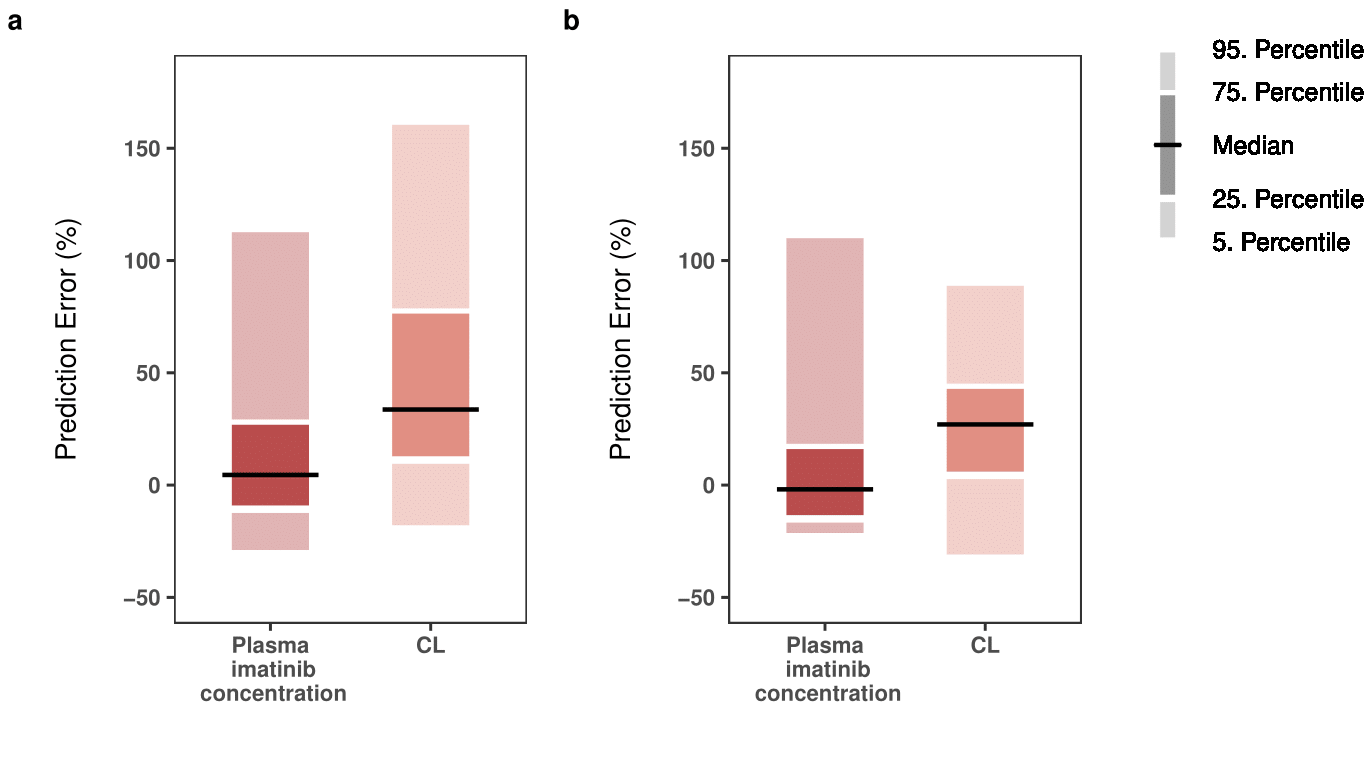


**Supplement Fig. S2** Distributions of the prediction errors

**(A)** all data **(B)** subset of the data within one dosing-interval

**Supplement Table S1** Demographic characteristics of study populations of healthy subject and patient

|  | Variable | Median (Range) |  | Variable | Count (Total) |
| --- | --- | --- | --- | --- | --- |
| Cohort of healthy subjects | | | | | |
|  | Body weight (kg) | 69.5 (52.0 – 96.0) |  | Gender (male) | 18 (26) |
|  | BMI (kg/m^2^) | 22.5 (20.0 – 30.0) |  | Race (Caucasian) | 26 (26) |
|  | Age | 23.0 (19.7 – 31.0) |  | Contraceptive Use | 4 (8) |
|  | Height (cm) | 175 (159 – 192) |  | Tobacco Consumption | 2 (26) |
|  | BSA (m^2^) | 1.86 (1.52 – 2.22) |  |  |  |
| Cohort of patients with GIST | | | | | |
|  | Body weight (kg) ^(1) (2)^ | 70.0 (43.0 – 98.0) |  | Gender (male) | 20 (40) |
|  | BMI (kg/m^2^) ^(1) (2)^ | 24.4 (18.4 – 32.4) |  | Gastric (yes) | 16 (40) |
|  | Age | 66.7 (35.0 – 83.2) |  |  |  |
|  | Height (cm) ^(2)^ | 165 (150 – 193) |  |  |  |
|  | BSA (m^2^) ^(1) (2)^ | 1.77 (1.34 – 2.13) |  |  |  |
| The information of contraceptive use was only collected from the eight female subjects.  ^(1)^ : documentation per administration  ^(2)^ : data for 3 patients missing | | | | | |

**Supplement Table S2** NONMEM models of imatinib published to date

| **Reference** | **N subject** | **N  sample** | **Indication** | **Structure model** | **Clearance [L/h] (IIV)** |
| --- | --- | --- | --- | --- | --- |
| Present Study | 26 | 472 | Healthy volunteers | 2 comp. 1.abs + 3.62 transit comp. | 13.2 (24.8%) |
| Park et al. (2016) ^[2]^ | 112 | 1773 | Healthy volunteers | 2 comp. Weibull absorption | 13.6 (23.4%) |
| Eechoute et al. (2012) ^[3]^ | 50 | 1743 | GIST | 2 comp. 1.abs +  5 transit comp. | 9.12 (49.5%) |
| van Erp et al. (2007) ^[4]^ | 11 | 198 | GIST | 1 comp. 1.abs | 9.4 (33%) |
| Widmer et al. (2006) ^[5]^ | 58 | 321 | CML/GIST | 1 comp. 1.abs | 14.3 (36%) |
| Haouala et al. (2012) ^[6]^ | 49 | 150 | GIST | 1 comp. 1.abs | 13.5 (23%) |
| Petain et al. (2008) ^[7]^ | 67 | 305 | GIST/  Solid malignancies | 1 comp. 1.abs | 7.29 (19%) |
| Schmidli et al. (2005) ^[8]^ | 371 | 1930 | CML | 1 comp. 0.abs | d1: 13.8 d29: 10.0 (31.9%) |
| Adeagbo et al. (2017) ^[9]^ | 126 | 250 | CML | 1 comp. 0.abs | 17.2 (17.4%) |
| Delbaldo et al. (2006) ^[10]^ | 35 | 166 | GIST | 1 comp. 0.abs | 7.97 (29%) |
| Judson et al. (2005) ^[11]^ | 42 | 517 | GIST/  Soft tissue sarcoma | 1 comp. 0.abs | d1: 9.23  d29: 8.71  ext.phase: 14.7 |
| Yamakawa et al. (2011) ^[12]^ | 34 | 626 | CML | 1 comp. 0.abs | 8.7 |
| Menon-Andersen et al. (2009) ^[13]^ | 41 | 842 | CML/GIST | 1 comp. 0.abs | 10.8 (31.5%) |
| Golabchifar et al. (2014) ^[14]^ | 61 | 533 | CML | 1 comp. 0.abs +  lag time | 10.8 (30%) |
| comp.: compartment; 1.abs: first order absorption; 0.abs: zero order absorption; IIV: inter-individual variability. GIST: Gastrointestinal stromal tumor, CML: Chronic myelogenous leukemia. d1: day 1 of imatinib treatment. d29: day 29 of imatinib treatment. ext.phase: extension phase. | | | | | |

# Reference

1. Pena M, Muriel J, Saiz-Rodríguez M, Borobia AM, Abad-Santos F, Frías J, et al. (2020) Effect of Cytochrome P450 and ABCB1 Polymorphisms on Imatinib Pharmacokinetics After Single-Dose Administration to Healthy Subjects. Clin Drug Investig 40:617-28. <https://doi.org/10.1007/s40261-020-00921-7>

2. Park G-j, Park W-S, Bae S, Park S-m, Han S, Yim D-S (2016) Population pharmacokinetics of imatinib mesylate in healthy Korean subjects. Transl Clin Pharmacol 24:96-104.

3. Eechoute K, Fransson MN, Reyners AK, de Jong FA, Sparreboom A, van der Graaf WT, et al. (2012) A long-term prospective population pharmacokinetic study on imatinib plasma concentrations in GIST patients. Clin Cancer Res 18:5780-7. <https://doi.org/10.1158/1078-0432.Ccr-12-0490>

4. van Erp NP, Gelderblom H, Karlsson MO, Li J, Zhao M, Ouwerkerk J, et al. (2007) Influence of CYP3A4 inhibition on the steady-state pharmacokinetics of imatinib. Clin Cancer Res 13:7394-400. <https://doi.org/10.1158/1078-0432.Ccr-07-0346>

5. Widmer N, Decosterd LA, Csajka C, Leyvraz S, Duchosal MA, Rosselet A, et al. (2006) Population pharmacokinetics of imatinib and the role of alpha-acid glycoprotein. Br J Clin Pharmacol 62:97-112. <https://doi.org/10.1111/j.1365-2125.2006.02719.x>

6. Haouala A, Widmer N, Guidi M, Montemurro M, Leyvraz S, Buclin T, et al. (2013) Prediction of free imatinib concentrations based on total plasma concentrations in patients with gastrointestinal stromal tumours. Br J Clin Pharmacol 75:1007-18. <https://doi.org/10.1111/j.1365-2125.2012.04422.x>

7. Petain A, Kattygnarath D, Azard J, Chatelut E, Delbaldo C, Geoerger B, et al. (2008) Population pharmacokinetics and pharmacogenetics of imatinib in children and adults. Clin Cancer Res 14:7102-9. <https://doi.org/10.1158/1078-0432.Ccr-08-0950>

8. Schmidli H, Peng B, Riviere GJ, Capdeville R, Hensley M, Gathmann I, et al. (2005) Population pharmacokinetics of imatinib mesylate in patients with chronic-phase chronic myeloid leukaemia: results of a phase III study. Br J Clin Pharmacol 60:35-44. <https://doi.org/10.1111/j.1365-2125.2005.02372.x>

9. Adeagbo B, Olugbade T, Durosinmi M, Bolarinwa R, Ogungbenro K, Bolaji O (2017) Population Pharmacokinetics of Imatinib in Nigerians With Chronic Myeloid Leukemia: Clinical Implications for Dosing and Resistance. Journal of clinical pharmacology 57. <https://doi.org/10.1002/jcph.953>

10. Delbaldo C, Chatelut E, Ré M, Deroussent A, Séronie-Vivien S, Jambu A, et al. (2006) Pharmacokinetic-pharmacodynamic relationships of imatinib and its main metabolite in patients with advanced gastrointestinal stromal tumors. Clin Cancer Res 12:6073-8. <https://doi.org/10.1158/1078-0432.Ccr-05-2596>

11. Judson I, Ma P, Peng B, Verweij J, Racine A, di Paola ED, et al. (2005) Imatinib pharmacokinetics in patients with gastrointestinal stromal tumour: a retrospective population pharmacokinetic study over time. EORTC Soft Tissue and Bone Sarcoma Group. Cancer Chemother Pharmacol 55:379-86. <https://doi.org/10.1007/s00280-004-0876-0>

12. Yamakawa Y, Hamada A, Nakashima R, Yuki M, Hirayama C, Kawaguchi T, et al. (2011) Association of genetic polymorphisms in the influx transporter SLCO1B3 and the efflux transporter ABCB1 with imatinib pharmacokinetics in patients with chronic myeloid leukemia. Ther Drug Monit 33:244-50. <https://doi.org/10.1097/FTD.0b013e31820beb02>

13. Menon-Andersen D, Mondick JT, Jayaraman B, Thompson PA, Blaney SM, Bernstein M, et al. (2009) Population pharmacokinetics of imatinib mesylate and its metabolite in children and young adults. Cancer Chemother Pharmacol 63:229-38. <https://doi.org/10.1007/s00280-008-0730-x>

14. Golabchifar AA, Rezaee S, Ghavamzadeh A, Alimoghaddam K, Dinan NM, Rouini MR (2014) Population pharmacokinetics of imatinib in Iranian patients with chronic-phase chronic myeloid leukemia. Cancer Chemother Pharmacol 74:85-93. <https://doi.org/10.1007/s00280-014-2473-1>
